# Mass spectrometry data from label-free quantitative proteomic analysis of harmless and pathogenic strains of infectious microalgae, *Prototheca spp*

**DOI:** 10.1016/j.dib.2017.04.006

**Published:** 2017-04-11

**Authors:** Jayaseelan Murugaiyan, Murat Eravci, Christoph Weise, Uwe Roesler

**Affiliations:** a Institute of Animal Hygiene and Environmental Health, Centre for Infectious Medicine, Freie Universitaet Berlin, Berlin, Germany; b Institute of Chemistry and Biochemistry, Freie Universitaet Berlin, Berlin, Germany

## Abstract

Here, we provide the dataset associated with our research article ‘label-free quantitative proteomic analysis of harmless and pathogenic strains of infectious microalgae, *Prototheca* spp.’ (Murugaiyan et al., 2017) [1]. This dataset describes liquid chromatography–mass spectrometry (LC–MS)-based protein identification and quantification of a non-infectious strain, *Prototheca zopfii* genotype 1 and two strains associated with severe and mild infections, respectively, *P. zopfii* genotype 2 and *Prototheca blaschkeae*. Protein identification and label-free quantification was carried out by analysing MS raw data using the MaxQuant-Andromeda software suit. The expressional level differences of the identified proteins among the strains were computed using Perseus software and the results were presented in [1]. This DiB provides the MaxQuant output file and raw data deposited in the PRIDE repository with the dataset identifier PXD005305.

**Specifications Table**

**Table:** 

Subject area	*Biology*
More specific subject area	*Label-free quantitative proteomics*, *Bovine mastitis-associated infectious microalgae, Prototheca. spp.*
Type of data	*Raw data,* table and Excel output files
How data was acquired	*LC-MS using an UltiMate 3000 HPLC system (Dionex) connected online to an LTQ-Orbitrap Velos (Thermo Scientific)*
Data format	*Raw, processed*
Experimental factors	a) *Cell culture, harvest and protein isolation* b) *In-solution trypsin digestion and mass spectrometry analysis* c) *Protein identification and quantitative proteomic analysis*
Experimental features	*Whole cell proteins were extracted from Prototheca cultured strains cultured until mid-logarithmic phase of growth.* *For each sample protein concentrations were determined using the Bradford assay (Bio-Rad). Proteins were reduced, alkylated and digested with trypsin in solution. Following LC–MS analysis, protein identification and quantification was performed with MaxQuant software, the label-free quantitation was carried out using Perseus software.*
Data source location	*Berlin, Germany*
Data accessibility	*Data available at PRIDE:*PXD005305.

**Value of the data**

• The data further validate the protein identification presented in Murugaiyan et al. [1].
• Data from the LC–MS analysis will provide researchers with detailed information on proteins associated with non-infectious, mildly and severely infectious strains of *Prototheca* spp.
• *Prototheca* spp. represents an “orphan species” whose genome sequence has not yet been sequenced, therefore, this raw data is useful for quick analysis once the genome sequence has become available.

## 1 Data

This mass spectrometry data-in-brief is associated with the research article aimed towards identification of differentially expressed proteins among three different strains of *Prototheca* spp., *Prototheca zopfii* genotype 1 (GT1), genotype 2 (GT2) and *Prototheca blaschkeae* [1]. The dataset comprises raw data, results of protein identification using MaxQuant-Andromeda software suit and a list of proteins identified as differentially expressed between non-infectious, infectious and mildly infectious strains of *Prototheca* spp. The raw data can be downloaded from the PRIDE repository (identifier PXD005305), a compilation of the identified proteins is presented in Supplementary table 1 and the differentially expressed proteins are listed in Table 1.

## 2 Experimental design

**Fig. 1:**
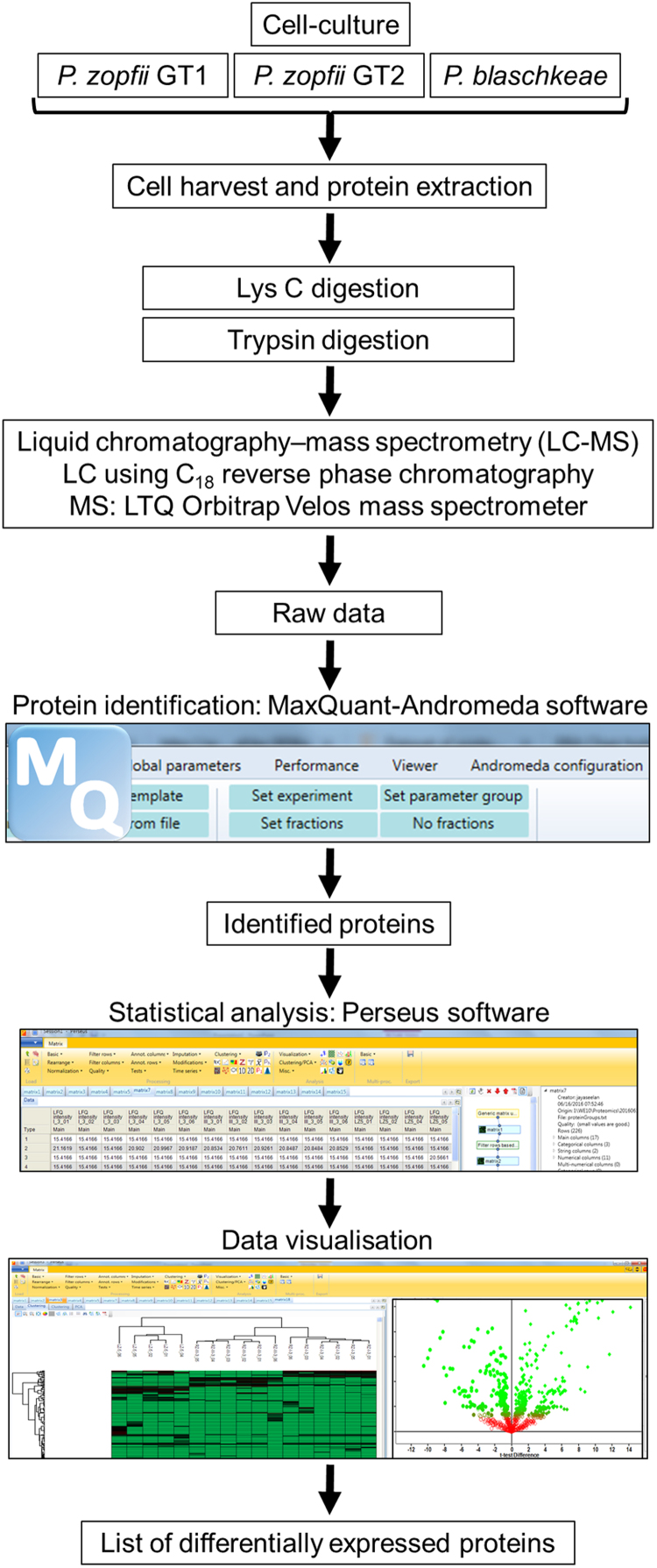
Schematic overview of the overall analysis workflow.

The dataset presented here was obtained from using the label-free proteomic analysis of three different strains of *Prototheca* species, *P. zopfii* genotype 1, genotype 2 and *P. blaschkeae* representing non-infectious, infectious and moderately infectious strains, respectively. In total 17 samples representing six independent cultures for each (only five in *P. zopfii* genotype 2) were used to generate the dataset (experimental design is shown in Fig. 1). A Student-*t* test, *p*-value <0.05% and 1% false discovery rate (FDR) was applied for identification of differentially expressed proteins between (a) *P. zopfii* genotype 2 and *P. zopfii* genotype 1; (b) *P. blaschkeae* and *P. zopfii* genotype 1; and (c) *P. zopfii* genotype 2 and *P. blaschkeae*.

## 3 Materials and methods

### 3.1 Prototheca strains

The following three strains from the culture collection of the Institute of Animal Hygiene and Environmental Health, Freie Universität Berlin, Germany were utilized for this study [3].

a. *P. zopfii* genotype 1 (SAG 2063^T^), non-infectious environmental strain.
b. *P. zopfii* genotype 2 (SAG 2021^T^), clinical strain.
c. *P. blaschkeae* (SAG 2064^T^), clinical strain.

### 3.2 Cell culture and protein extraction

Following the retrieval from the culture collection, the strains were first streaked in Sabouraud dextrose solid media, incubated at 37 °C until the appearance of visible colonies. The species and genotypes were reconfirmed using MALDI profiling as described [4]. The cell culture and protein extraction was carried out as described [1].

### 3.3 Mass spectrometry analysis

The proteins were subjected to in-solution trypsin digested as described [1]. The resultant peptides were purified using solid phase extraction procedure [5], separated by nanoscale C_18_ reverse-phase liquid chromatography using the Dionex Ultimate 3000 nanoLC (Dionex/Thermo Fisher Scientific, Idstein, Germany) and directly ionised by electrospray ionization and measured after transfer into an LTQ Orbitrap Velos mass spectrometer (Thermo Fisher Scientific, Bremen, Germany). MS survey scan (*m*/*z* 300–1700, resolution 60,000) was acquired in the Orbitrap and the 20 most intensive precursor ions were fragmented.

### 3.4 Data analysis

Data from MS/MS spectra was searched using MaxQuant-Andromeda software suit [6], [7], [8] against the Uniprot FASTA dataset of *Chlorella variabilis* and *Auxenochlorella protothecoides* proteome with the parameters settings as described in [1]. Table 2 shows the experimental design and sample file naming format and the dataset associated to the MaxQuant analysis is shown in Supplementary table 2.

The statistical analysis was carried out using Perseus 1.4.1.3 (Available online: http://141.61.102.17/ perseus_doku/doku.php?id=start) as described [1]. The differences in protein expression computed in three different ways i) mildly infectious vs environmental strain, ii) severe infection-associated vs environmental strain and iii) severely infectious vs mildly infectious strain were presented in Murugaiyan et al. [1].

### 3.5 Mass Spectrometry dataset deposit

The mass spectrometry data was deposited at the ProteomeXchange (PX) Consortium [9], [10], [11] via the PRIDE (PRoteomics IDEntifications) partner repository at the European Bioinformatics Institute (http://www.ebi.ac.uk/pride/) and is now accessible with the dataset identifier PXD005305.

## Figures and Tables

**Table 1:** List of proteins identified as differentially expressed.

**S.No**	**UniProt Acc. No.**	**Protein name**	−Log2(fold change)		
			*P. zopfii* GT2 vs *P. zopfii* GT1	*P. blaschkeae vs P. zopfii* GT1	*P. zopfii* GT2 vs *P. blaschkeae*
1	E1ZQV2	Heat shock protein 70	−1.0⁎	−0.4⁎	−0.6⁎
2	E1ZLA8	Acetyl-coenzyme A synthetase	−6.8⁎	−6.8⁎	0.0
3	A0A087SCT6	Citrate synthase	−3.6⁎	−3.6⁎	0.0
4	E1ZL24	Putative uncharacterized protein	−4.6⁎	−4.6⁎	0.0
5	A0A087SSM0	Actin	−0.6⁎	+0.1	−0.7⁎
6	A0A087SFG0	Cysteine synthase, chloroplastic/chromoplastic	−3.9⁎	+1.7	−5.6⁎
7	A0A087SP16	FK506-binding protein 1	−1.4⁎	−0.1	−1.3⁎
8	E1ZK88	Ubiquitin	−1.1⁎	+0.3	−1.4⁎
9	A0A087SJV3	Aldehyde dehydrogenase family 2 member B4	+0.5⁎	−0.5⁎	+1.0⁎
10	E1ZG37	Putative uncharacterized protein	+0.6⁎	−3.8⁎	+4.4⁎
11	A0A087SS91	Aconitate hydratase, mitochondrial (Aconitase)	+0.6⁎	−7.3⁎	+8.0
12	E1ZTB0	Fructose-bisphosphate aldolase	+8.3⁎	+8.8⁎	−0.6⁎
13	E1ZCI5	Putative uncharacterized protein	+0.5⁎	+0.7⁎	−0.3
14	E1ZT42	V-type H+ ATPase subunit A	+0.5⁎	+0.4⁎	+0.1
15	A0A087SJM7	40S ribosomal protein S10	+6.9⁎	0.0	+6.9⁎
16	E1ZQY4	40S ribosomal protein S5	+3.3⁎	0.0	+3.3⁎
17	A0A087SBU8	5-methyltetrahydropteroyltriglutamate-homocysteine methyltransferase	+6.4⁎	0.0	+6.4⁎
18	A0A087SNV1	60S ribosomal protein L12-1	+6.7⁎	0.0	+6.7⁎
19	A0A087SKG6	60S ribosomal protein L6	+4.4⁎	0.0	+4.4⁎
20	A0A087SN43	6-phosphogluconate dehydrogenase, decarboxylating (EC 1.1.1.44)	+4.5⁎	+0.7	+3.8⁎
21	A0A087SJX6	Argininosuccinate synthase	+3.6⁎	0.0	+3.6⁎
22	A0A087SPA9	Carbamoyl-phosphate synthase large chain	+4.6⁎	+1.1	+3.4⁎
23	A0A087SHS8	Eukaryotic initiation factor 4A-10	+0.4⁎	−0.2	+0.6⁎
24	E1ZFZ5	Glutamate dehydrogenase	+3.1⁎	0.0	+3.1⁎
25	A0A087SQ68	Phosphate carrier protein, mitochondrial	+3.1⁎	0.0	+3.1⁎
26	E1ZGA3	40S ribosomal protein S27	+3.3⁎	+1.2	+2.1
27	E1Z7R4	Heat shock protein 70	+5.3⁎	+2.2	+3.1
28	E1ZSM6	Putative uncharacterized protein	+3.3⁎	+1.2	+2.1
29	A0A087SF19	Adenosylhomocysteinase	+1.7	−2.4⁎	+4.2⁎
30	A0A087SK74	Elongation factor 1-alpha	+0.2	−0.6⁎	+0.8⁎
31	E1Z5R3	Putative uncharacterized protein	−1.6	−5.3⁎	+3.8⁎
32	E1ZJM1	Tubulin beta chain	0.0	−0.6⁎	+0.6⁎
33	A0A087SE71	Elongation factor Tu	−1.5	−4.3⁎	+2.8
34	A0A087SG29	Glucose-6-phosphate isomerase	−3.2	−5.3⁎	+2.1
35	A0A087SSF2	Nucleoside diphosphate kinase 1	−2.0	−4.5⁎	+2.5
36	A0A087SL21	Ubiquitin-60S ribosomal protein L40-2	−3.7	−8.2⁎	+4.5
37	A0A087SI38	Acetyl-coenzyme A synthetase	0.0	+4.6⁎	−4.6⁎
38	A0A087SBN0	ATP synthase subunit beta (Delta-aminolevulinic acid dehydratase)	0.0	+0.5⁎	−0.5⁎
39	A0A087SQR3	Chaperonin CPN60, mitochondrial	+0.2	+0.9⁎	−0.7⁎
40	A0A087SBQ6	Glyceraldehyde-3-phosphate dehydrogenase, cytosolic	0.0	+6.8⁎	−6.8⁎
41	A0A087SND2	Heat shock 70 kDa protein, mitochondrial	−0.1	+0.6⁎	−0.7⁎
42	A0A087ST26	Phosphoglycerate kinase	0.0	+5.5⁎	−5.5⁎
43	A0A087SNN6	Stress-induced-phosphoprotein 1	0.0	+3.7⁎	−3.7⁎
44	A0A087SIY9	Succinyl-CoA ligase [ADP-forming] subunit alpha-1, mitochondrial	0.0	+4.7⁎	−4.7⁎
45	A0A087S9W3	Histone H4	0.0	+2.9⁎	−2.9
46	E1ZRV3	Putative uncharacterized protein	+0.7	+4.3⁎	−3.6
47	E1ZMD2	Putative uncharacterized protein	0.0	+2.4⁎	−2.4
48	A0A087SAK4	Chaperone protein ClpB1	−0.8	+2.0	−2.8⁎
49	A0A087S9L8	Enolase	−3.7	+1.7	−5.4⁎
50	A0A087SI84	GTP-binding nuclear protein	−0.6	+0.4	−1.0⁎
51	E1ZD41	Putative uncharacterized protein	+3.3	−0.7	+4.0⁎

(+) indicates upregulated and (−) indicates downregulated.

⁎ Statistical significance was calculated using two-way Student-*t* test and error correction (*p* value <0.05) using the method of Benjamini–Hochberg [2].

**Table 2:** Experimental design and raw data file naming format.

**S. No**	**Sample name**	**Strain designation**	**Replicates**	**raw data file designation**
1	*P. zopfii* genotype 1	SAG 2063^T^	1	I_3_01
2			2	I_3_02
3			3	I_3_03
4			4	I_3_04
5			5	I_3_05
6			6	I_3_06
				
7	*P. blaschkeae*	SAG 2064^T^	1	III_3_01
8			2	III_3_02
9			3	III_3_03
10			4	III_3_04
11			5	III_3_05
12			6	III_3_06
				
13	*P. zopfii* genotype 2	SAG 2021^T^	1	LZ5_01
14			2	LZ5_02
15			3	sample lost during transit
16			4	LZ5_04
17			5	LZ5_05
18			6	LZ5_06
